# Supplementary material for: Impaired liver regeneration and lipid homeostasis in CCl4 treated WDR13 deficient mice
Source: Lab Anim Res. 2020 Nov 13;36:41. doi: 10.1186/s42826-020-00076-8 (PMC7666495; doi:10.1186/s42826-020-00076-8)
Supplement: Supplementary file 1 — Additional file 1 [file 42826_2020_76_MOESM1_ESM.pdf]

Impaired liver regeneration and lipid homeostasis in CCl_4 treated WDR13 deficient mice

Arun Prakash Mishra[1,2*], Archana B Siva[1], Chandrashekaran Gurunathan[1], Komala Y[1], B Jyothi Lakshmi[1]
[1]CSIR- Centre for Cellular and Molecular Biology, Hyderabad -500007, India
[2]National Cancer Institute, NIH, Frederick MD-21702, USA

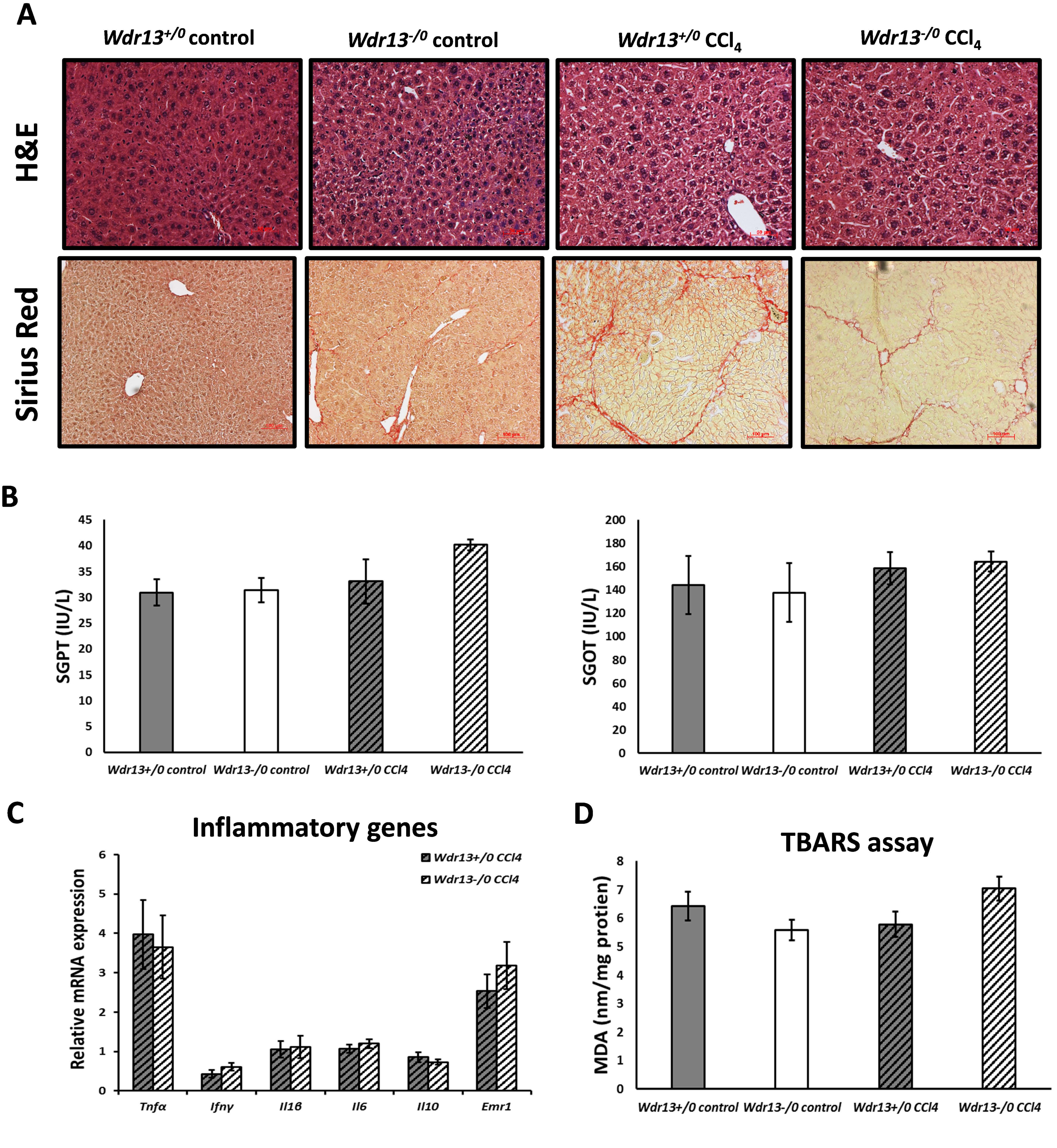

Supplementary Fig. S1. Intensity of liver damage. A) Liver morphology is depicted using H&E staining and collagen deposition in liver by Sirius red staining. B) SGPT and SGOT analyses of serum. C) mRNA levels of genes involved in inflammation. D) TBARS analysis of the liver. n=5 mice for controls and n=8 mice for treatment.
